# The Short-term Prognostic Value of the Triglyceride-to-high-density Lipoprotein Cholesterol Ratio in Acute Ischemic Stroke

**DOI:** 10.14336/AD.2017.0629

**Published:** 2018-06-01

**Authors:** Qi-Wen Deng, Shuo Li, Huan Wang, Leix Lei, Han-Qing Zhang, Zheng-Tian Gu, Fang-Lan Xing, Fu-Ling Yan

**Affiliations:** Department of Neurology, Affiliated ZhongDa Hospital, School of Medicine, Southeast University, Nanjing, China; Department of Neurology, Affiliated ZhongDa Hospital, School of Medicine, Southeast University, Nanjing, China; Department of Neurology, Affiliated ZhongDa Hospital, School of Medicine, Southeast University, Nanjing, China; Department of Neurology, Affiliated ZhongDa Hospital, School of Medicine, Southeast University, Nanjing, China; Department of Neurology, Affiliated ZhongDa Hospital, School of Medicine, Southeast University, Nanjing, China; Department of Neurology, Affiliated ZhongDa Hospital, School of Medicine, Southeast University, Nanjing, China; Department of Neurology, Affiliated ZhongDa Hospital, School of Medicine, Southeast University, Nanjing, China; Department of Neurology, Affiliated ZhongDa Hospital, School of Medicine, Southeast University, Nanjing, China

**Keywords:** triglyceride, high-density lipoprotein cholesterol, TG/HDL-C, acute ischemic stroke, survival

## Abstract

The triglyceride (TG)-to-high-density lipoprotein cholesterol (HDL-C) ratio (TG/HDL-C) is a simple approach to predicting unfavorable outcomes in cardiovascular disease. The influence of TG/HDL-C on acute ischemic stroke remains elusive. The purpose of this study was to investigate the precise effect of TG/HDL-C on 3-month mortality after acute ischemic stroke (AIS). Patients with AIS were enrolled in the present study from 2011 to 2017. A total of 1459 participants from a single city in China were divided into retrospective training and prospective test cohorts. Medical records were collected periodically to determine the incidence of fatal events. All participants were followed for 3 months. Optimal cutoff values were determined using X-tile software to separate the training cohort patients into higher and lower survival groups based on their lipid levels. A survival analysis was conducted using Kaplan-Meier curves and a Cox proportional hazards regression model. A total of 1459 patients with AIS (median age 68.5 years, 58.5% male) were analyzed. Univariate Cox regression analysis confirmed that TG/HDL-C was a significant prognostic factor for 3-month survival. X-tile identified 0.9 as an optimal cutoff for TG/HDL-C. In the univariate analysis, the prognosis of the TG/HDL-C >0.9 group was markedly superior to that of TG/HDL-C ≤0.9 group (P<0.001). A multivariate Cox regression analysis showed that TG/HDL-C was independently correlated with a reduced risk of mortality (hazard ratio [HR], 0.39; 95% confidence interval [CI], 0.24-0.62; P<0.001). These results were confirmed in the 453 patients in the test cohort. A nomogram was constructed to predict 3-month case-fatality, and the c-indexes of predictive accuracy were 0.684 and 0.670 in the training and test cohorts, respectively (P<0.01). The serum TG/HDL-C ratio may be useful for predicting short-term mortality after AIS.

Stroke is a leading cause of mortality and disability in adults resulting in a substantial social burden worldwide [1]. More than 80% of patients with a history of stroke have experienced a cerebral arterial occlusion leading to brain ischemia. Stroke severity is the most useful predictor of stroke outcome [2-5]. Additionally, age, cardiac disease, stroke etiology, diabetes, and hypertension are important prognostic factors of long-term outcomes [2-5]. The identification of modifiable prognostic factors in acute ischemic stroke (AIS) allows physicians to select an appropriate treatment to improve prognoses. However, despite the available diagnosis and treatment strategies, stroke mortality has not significantly improved over the past four decades [6].

Hyperlipidemia is a well-documented risk factor for cardiovascular disease [7]. However, previous observational studies attempting to explain the influence of the serum lipid level on stroke outcome have reported mixed findings [8-12]. Higher values of the triglyceride (TG)-to-high-density lipoprotein cholesterol (HDL-C) ratio (TG/HDL-C) values are associated with early neurological deterioration, whereas lower ratios are associated with early clinical improvement [12]. Our previous study demonstrated that a lower TG/HDL-C is independently correlated with mortality and poor prognosis in AIS patients [13]. Nevertheless, how TG/HDL-C can facilitate the clinical management of AIS patients in daily practice remains to be addressed.

The present study enrolled two cohorts to investigate the precise effect of the TG/HDL-C ratio on 3-month mortality after AIS, and we observed that a lower TG/HDL-C value was significantly associated with 3-month mortality in the training and test cohort patients. Additionally, there are many reports on the successful establishment of nomograms for disease prognostics, but nomograms for predicting stroke outcome are scarce. Therefore, we evaluated the predictive value of a nomogram based on TG/HDL-C in patients with AIS.

**Table 1:** Baseline bivariate comparison of training and test cohort.

Demographic characteristics	Training cohort Total	Incident alive	Incident death	*P* value^a^	Test cohort Total	Incident death		*P*-value^a^
Age	68.5 (57-77)	68 (57-76)	72 (60-82)	<0.001	69 (56-76)	68.5 (56-76)	71 (59-83)	<0.001
Gender (male/female)	585/421	512/385	73/36	0.048	268/185	238/174	30/11	0.056
BMI (kg/m^2^)	23.6 (22.0-24.9)	23.6 (21.8-24.9)	24.2 (22.4-25.0)	0.219	23.2 (21.8-25.4)	23.2 (21.7-25.4)	23.9 (22.0-25.8)	0.096
Clinical characteristics								
Smoking (yes/no)	245/761	212/685	33/76	0.127	118/335	102/310	16/25	0.047
Baseline NIHSS	6 (3-10)	6 (3-10)	6 (2.75-10)	0.014	9 (3-17)	8 (3-16)	9 (3-17)	0.005
Therapy of thrombolysis								
Present	101 (10.0)	97 (10.8)	4 (3.7)	0.030	35 (7.7)	33 (8.0)	2 (4.9)	0.474
Absent	905 (90.0)	800 (89.2)	105 (96.3)		418 (92.3)	379 (92.0)	39 (95.1)	
Endovascular intervention								
Present	42 (4.2)	40 (4.5)	2 (1.8)	0.196	13 (2.9)	12 (2.9)	1 (2.4)	0.862
Absent	964 (95.8)	857 (95.5)	107 (98.2)		440 (97.1)	400 (97.1)	40 (97.6)	
Laboratory characteristics								
TG (mmol/L)	1.34 (1.02-1.96)	1.37 (1.04-2.00)	1.13 (0.81-1.48)	<0.001	1.30 (1.01-1.96)	1.34 (1.01-2.00)	1.22 (0.83-1.65)	0.004
TC (mmol/L)	4.85 (4.19-5.53)	4.88 (4.24-5.53)	4.44 (3.94-5.44)	0.022	4.88 (4.19-5.55)	4.91 (4.20-5.67)	4.44 (4.08-5.36)	0.108
HDL-C (mmol/L)	1.23 (1.10-1.47)	1.23 (1.10-1.46)	1.29 (1.13-1.57)	0.041	1.25 (1.08-1.48)	1.25 (1.08-1.47)	1.31 (1.13-1.55)	0.186
LDL-C (mmol/L)	2.86 (2.48-3.41)	2.89 (2.50-3.43)	2.72 (2.33-3.28)	0.017	2.87 (2.51-3.47)	2.90 (2.51-3.47)	2.79 (2.51-3.42)	0.106
TG/HDL-C	1.06 (0.78-1.61)	1.09 (0.81-1.65)	0.79 (0.65-1.08)	<0.001	1.04 (0.77-1.59)	1.08 (0.78-1.64)	0.84 (0.65-1.14)	0.007

BMI, body mass index; NIHSS, NIH Stroke Scale; Triglycerides; TC, total cholesterol; HDL-C, high-density lipoprotein cholesterol; LDL-C, low-density lipoprotein cholesterol; TG/HDL-C, TG to HDL-C ratio Values are medians (interquartile range) or frequencies and percentages. Statistically significant results were in bold.

a χ^2^ test or Mann-Whitney *U* test.

## MATERIALS AND METHODS

### Patients

We acquired data from the Affiliated ZhongDa Hospital of Southeast University and from Nanjing First Hospital of Nanjing Medical University. The baseline characteristics of the two cohorts are listed in Tables 1 and S1. Biomarkers were retrospectively available from 1006 individuals with symptoms of AIS before statin treatment in the training cohort; the training cohort patients were obtained from our previous study [13]. An additional 453 AIS patients were prospectively recruited in the test cohort. The follow-up period for the AIS patients was 3 months. Individual patients were enrolled in the current study if they met the following criteria: (1) first onset of stroke; (2) blood sample extraction within 36 hours of stroke onset; (3) confirmed ischemic stroke; and (4) performance of standard in-house procedures. Several individuals were excluded based on the following criteria: (1) consuming more than 40 g of alcohol per day, which may influence TG levels, and (2) being pre-stroke with severe infectious disease or malignancies. Clinical outcomes, including whether the patients were alive or dead, were further assessed after 3 months. Each participant was followed up after 3 months via telephone, email, and contact in an outpatient clinic. The study protocol was approved by the Southeast University Ethics Committee and complied with the Declaration of Helsinki.

### In-house procedures

All in-house procedures were performed as described in our study [13].

**Table 2:** Univariable analysis of lipid levels as continuous variables on 3-month mortality in the training cohort.

Variable	Hazard ratio	95% confidence interval	*P* value
TG	0.58	0.43-0.79	<0.001
TC	0.82	0.68-0.98	0.031
HDL-C	1.77	1.00-3.15	0.052
LDL-C	0.72	0.55-1.23	0.112
TG/HDL-C	0.44	0.30-0.66	<0.001

TG, Triglycerides; TC, total cholesterol; HDL-C, high-density lipoprotein cholesterol; LDL-C, low-density lipoprotein cholesterol; TG/HDL-C, TG to HDL-C ratio.

Statistically significant results were in bold.

### Clinical and laboratory evaluation

Stroke severity was assessed at admission using the National Institutes of Health Stroke Scale (NIHSS). Stroke etiology was determined using the modified Trial of Org 10172 in Acute Stroke Treatment (TOAST) classification. The treatment followed the guidelines of the American Heart Association and American Stroke Association [14]. Additionally, some AIS patients with NIHSS scores greater than 4 received the thrombolysis therapy and endovascular intervention.

Clinical and laboratory data were obtained from the medical records of individual patients. We recorded the following parameters: (1) demographic characteristics (age, gender, body mass index); (2) clinical characteristics (smoking, baseline NIHSS, and blood pressure); (3) medical history (hypertension, diabetes mellitus, atrial fibrillation, and transient ischemic attack); (4) stroke etiology; (5) thrombolysis therapy; (6) endovascular intervention therapy; and (7) laboratory characteristics, including white blood cell count (WBC), platelets, serum glucose concentration, creatinine, TG, total cholesterol (TC), HDL-C, and low-density lipoprotein cholesterol (LDL-C).

### Statistical analysis

The TG/HDL-C value was calculated by dividing TG by HDL-C. All data are expressed as frequencies and percentages for categorical variables and medians and interquartile ranges for continuous variables. Continuous variables and categorical variables were analyzed using rank sum tests (Mann-Whitney *U* tests) and chi-squared tests, respectively. We utilized X-tile software (Yale University, New Haven, USA) to determine the optimal cutoff values of TG, TC, and TG/HDL-C. The discriminatory abilities of these variables were determined via receiver operating characteristic (ROC) curves. Three-month survival was analyzed with a Kaplan-Meier curve and log-rank test. Univariable Cox regression analysis was used to investigate the prognostic significance of continuous and categorical lipid measures in AIS patients. Significant predictors in the univariable analysis were included in a multivariable regression model to determine independent predictors. Additionally, a nomogram and calibration curve was established using R for Windows with the package *rms* [15]. The discriminatory ability of the nomogram was evaluated using Harrell’s c-index. Calibration was used to compare the actual 3-month survival with the predicted probability predicted by the curve. Statistical analysis was performed with SPSS software (Version 20, Chicago, USA). A *P* value less than 0.05 was considered statistically significant.

**Figure 1.**
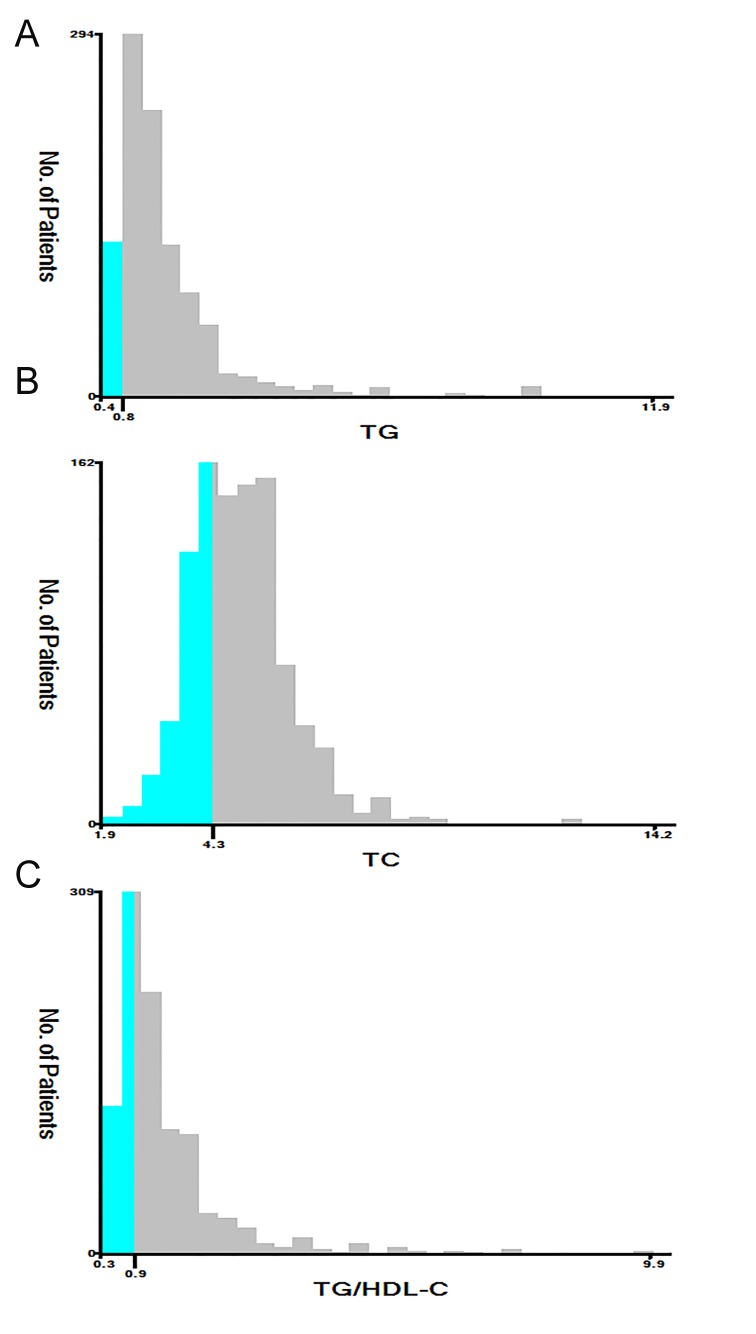
Analysis of TG (A), TC (B), and TG/HDL-C (C) using X-tile. The black circles highlight the optimal cutoff values which are presented in histograms.

**Figure 2.**
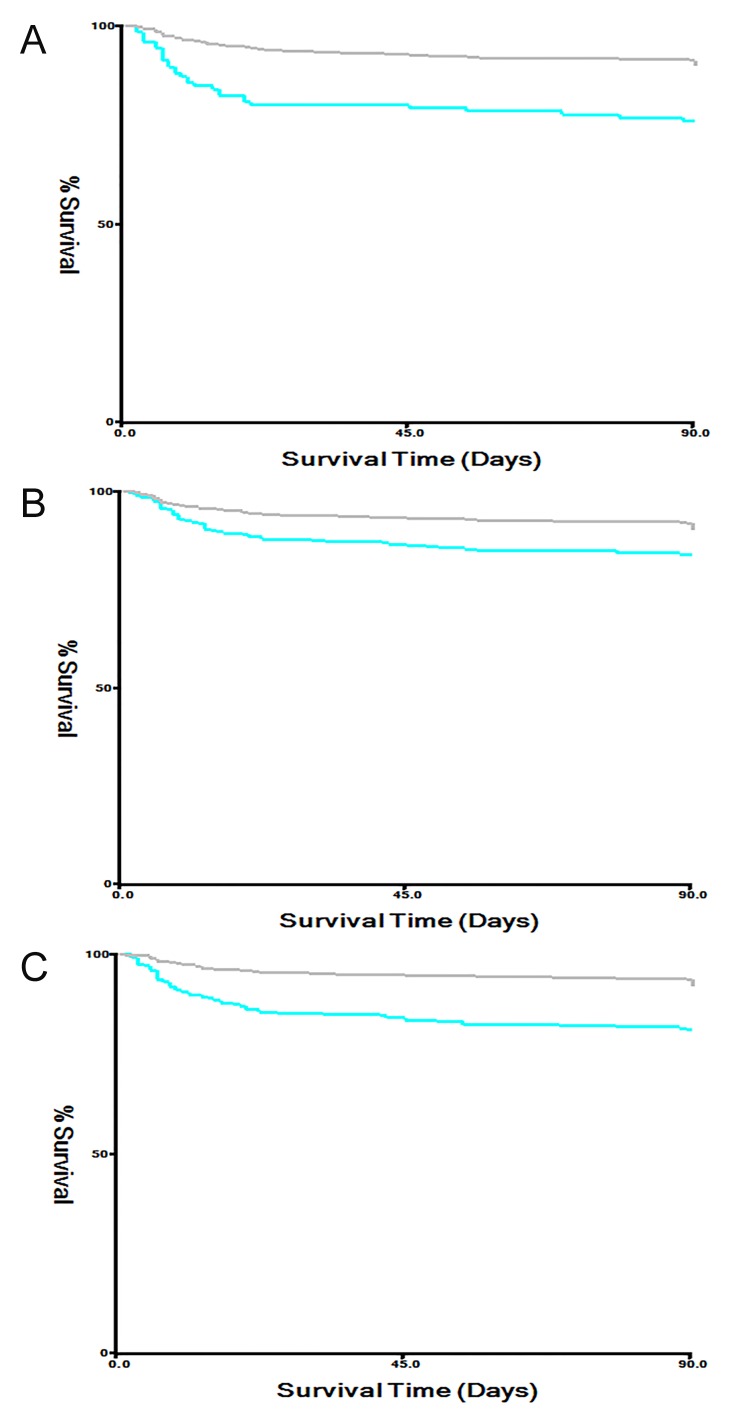
Survival curves of TG, TC, and TG/HDL-C in the training cohort. The low TG (A), TC (B), and TG/HDL-C (C) groups had higher 3-month mortality rates than the high groups in AIS patients (*P*<0.01).

## RESULTS

### Baseline characteristics

The baseline characteristics of the training and test cohorts of the present study are summarized in Tables 1 and S1. A total of 585 men and 421 women were retrospectively recruited into the training cohort. Of these patients, 897 (89.2%) were still alive after 3 months. Baseline demographics and the median concentrations of TG, TC, HDL-C, and LDL-C, as well as TG/HDL-C, are shown in Table 1. Data from an additional 453 patients were prospectively collected for the test cohort. Of these patients, 35 (7.7%) individuals received thrombolysis, and 13 (2.9%) experienced endovascular intervention. Forty-one died during the follow-up period. We also confirmed that old age, high NIHSS, low TG, and low TG/HDL-C were associated with an increased risk of mortality in the training and test cohort patients (*P*<0.05, Table 1).

The baseline characteristics of the training and test cohorts of the present study are summarized in Table 1 and Supplemental Table 1. A total of 585 men and 421 women were retrospectively recruited into the training cohort. Of these patients, 897 (89.2%) were still alive after 3 months. Baseline demographics and the median concentrations of TG, TC, HDL-C, and LDL-C, as well as TG/HDL-C, are shown in Table 1. Data from an additional 453 patients were prospectively collected for the test cohort. Of these patients, 35 (7.7%) individuals received thrombolysis, and 13 (2.9%) experienced endovascular intervention. Forty-one died during the follow-up period. We also confirmed that old age, high NIHSS, low TG, and low TG/HDL-C were associated with an increased risk of mortality in the training and test cohort patients (*P*<0.05, Table 1).

### Association of lipid level with survival

The TG, TC, HDL-C, LDL-C, and TG/HDL-C values were used as continuous variables, and univariable Cox regression analyses showed that TG, TC, and TG/HDL-C were significant prognostic factors in the training cohort (Table 2). X-tile was employed to determine the optimal cutoff values; in terms of 3-month mortality in the training cohort patients, these cutoff values were 0.8 μmol/l for TG, 4.3 μmol/l for TC, and 0.9 for TG/HDL-C (Fig. 1). The training cohort individuals were then divided into low- and high-level groups according to these cutoffs. We first compared the clinical characteristics of the high and low-level groups in training and test cohort patients. In the training cohort, differences between the high and low TG groups were observed for gender, smoking, medical history, stroke etiology, WBC, glucose, and creatinine (P<0.05). Additionally, TG/HDL-C was associated with gender, smoking, hypertension, and medical history (except history of TIA) (P<0.05). However, the differences between the two TC groups were not significant for any characteristic (Supplemental Table 2). Similar findings were observed when the test cohort patients were divided into low- and high-level groups for clinical characteristics according to the same cutoff values as the training cohort (Supplemental Table 3).

The univariate log-rank test results for clinical characteristics in the training and test cohorts are shown in Table 3. The low TG, TC, and TG/HDL-C groups had higher 3-month mortality rates than the high groups in AIS patients (P<0.01, Figure 2). A multivariate Cox regression analysis was then performed for clinical variables identified as significant in the univariate log-rank test. TG, TC, and TG/HDL-C, as well as age and hypertension, were independent prognostic factors for 3-month mortality in both the training and test cohort patients (Table 3).

**Table 3:** Univariate and multivariate analyses for the potential prognostic variables associated with 3-month mortality.

	Training cohort			Test cohort		

Variable	Univariate analysis	Multivariate analysis		Univariate analysis	Multivariate analysis	
	*P* value	HR (95%CI)	*P* value	*P* value	HR (95%CI)	*P* value
Age	<0.001	1.03 (1.02-1.05)	<0.001	0.008	1.18 (1.01-2.00)	0.008
Gender (female)	0.049	0.86 (0.57-1.30)	0.859	0.060		
BMI	0.476			0.142		
Smoking	0.135			0.050		
Baseline NIHSS	0.255			0.701		
SBP	0.057			0.074		
DBP	0.536			0.939		
Hypertension	0.019	1.85 (1.09-3.16)	0.023	0.013	1.68 (1.10-5.51)	0.030
Diabetes mellitus	0.268			0.621		
History of AT	0.091			0.807		
History of TIA	0.606			0.123		
Stroke etiology	0.449			0.757		
Therapy of thrombolysis	0.154			0.586		
Endovascular intervention	0.148			0.841		
WBC	0.621			0.918		
Platelet	0.724			0.519		
Glucose	0.072			0.986		
Creatinine	0.722			0.657		
TG	<0.001	0.42 (0.26-0.60)	<0.001	0.017	0.47 (0.22-0.73)	<0.001
TC	0.001	0.55 (0.38-0.81)	<0.001	0.026	0.50 (0.28-0.90)	0.001
HDL-C	0.089			0.167		
LDL-C	0.054			0.696		
TG/HDL-C	<0.001	0.39 (0.24-0.62)	<0.001	0.010	0.28 (0.12-0.65)	<0.001

BMI, body mass index; NIHSS, NIH Stroke Scale; SBP, systolic blood pressure; DBP, diastolic blood pressure; AT, atrial fibrillation; TIA, transient ischemic attack; WBC, white blood cell; TG, Triglycerides; TC, total cholesterol; HDL-C, high-density lipoprotein cholesterol; LDL-C, low-density lipoprotein cholesterol; TG/HDL-C, TG to HDL-C ratio.

Individual patients were divided into low (<cutoff value) and high (≥cutoff value) level groups according to corresponding cutoffs of serum lipids.

Statistically significant results were in bold.

### Predictive values of TG, TC, and TG/HDL-C for survival

**Figure 3.**
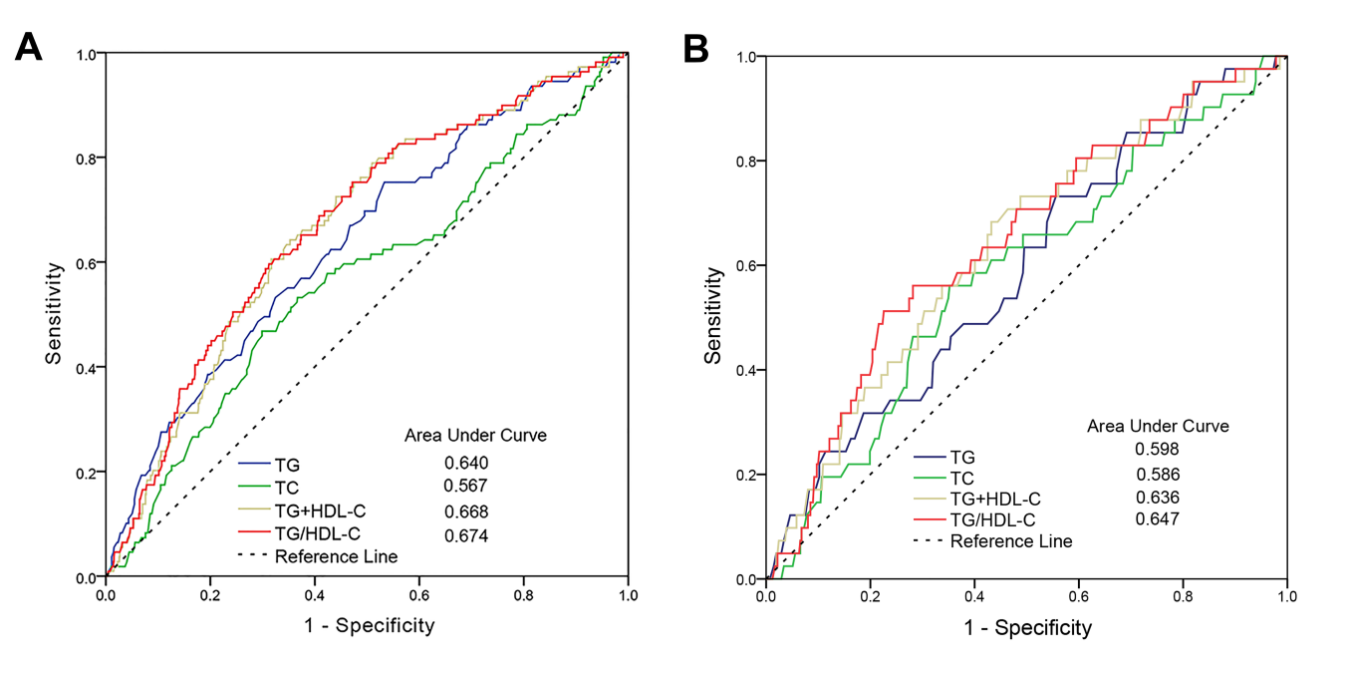
Predictive values of TG, TC, and TG/HDL-C for 3-month mortality in the training (A) and the test (B) cohort.

To investigate the predictive values of TG, TC, and TG/HDL-C in AIS patients, a ROC curve and areas under the curves (AUCs) regarding 3-month mortality were plotted and analyzed for the training cohort (Figure 3A). Moreover, we calculated the AUC of the combination of TG and HDL-C. The results showed that the optimal predictive value of TG/HDL-C (AUC=0.674) was significantly (AUC=0.640), TC (AUC=0.567), and their combined effect (AUC=0.668). Additionally, similar results were obtained when their predictive values were further confirmed by the ROC curve and AUC analyses in the test cohort (Figure 3B).

### New prognostic model

**Figure 4.**
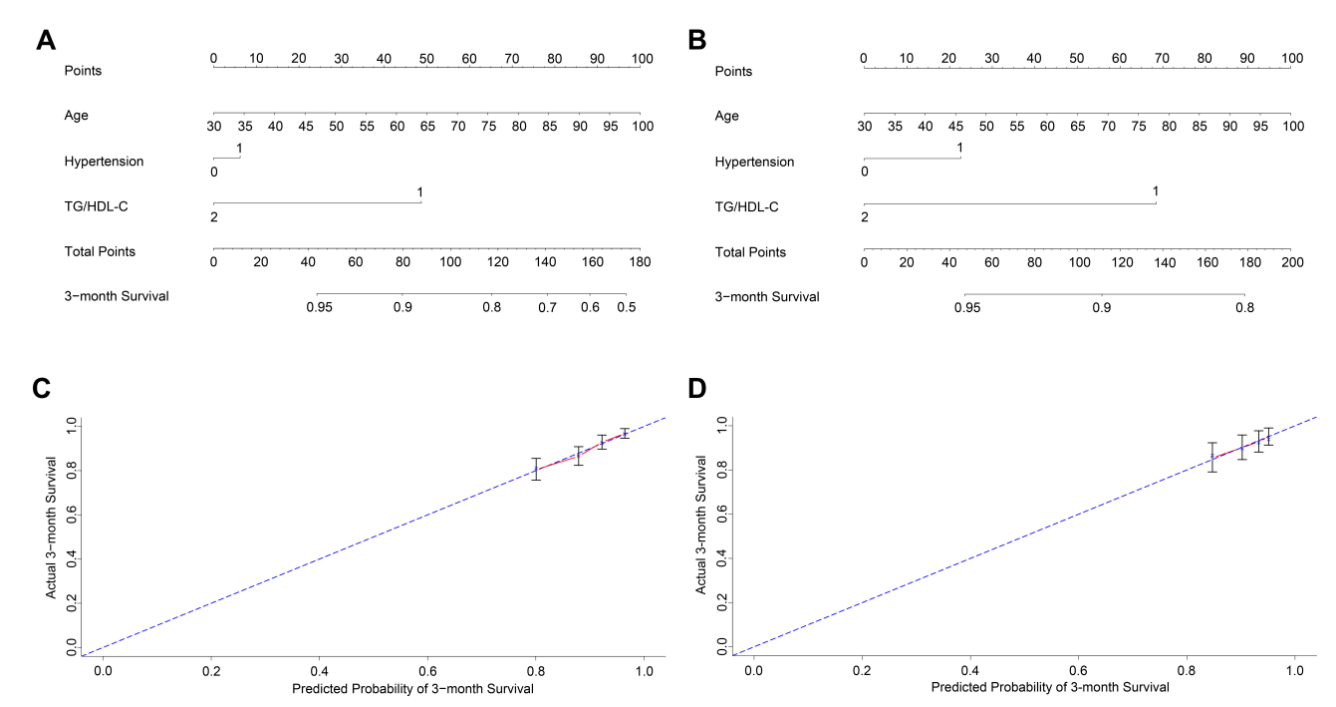
Nomogram of the study population to predict 3-month survival in acute ischemic stroke. The nomogram is used by summing the points assigned to the corresponding factors, which are presented at the top of the scale. The total is used to predict the 3-month probability of survival in the lowest scale. The c-indexes values for the training (A) and the test cohort (B) are 0.684 and 0.670, respectively. Individual patients were divided into low (<0.90)- and high (≧0.90)-level groups according to the TG/HDL-C cutoff. Calibration curves for 3-month survival, which are representative of predictive accuracy, for the training (C) and the test cohort (D). The 45-degree reference line represents a perfect match between predicted and observed values.

To further evaluate the predictive ability of TG/HDL-C in AIS patients, we used a nomogram based on the findings from the univariate analyses to predict 3-month mortality. The univariable analyses confirmed that age, history of hypertension, TG, TC, and TG/HDL-C (P<0.05) were significantly correlated with survival outcome in the training and test cohort patients. Among lipid classifications, the TG/HDL-C had the greatest AUC, and TG/HDL-C, age, and history of hypertension were included in the predictive model for both the training (Figure 4A) and test (Figure 4B) cohorts; R software was used with stepwise Cox regression analyses. The nomograms for both cohorts showed that old age and a history of hypertension were indicators of a poor prognosis, whereas a high TG/HDL-C was a favorable factor. These results were similar to those acquired previously in the multivariate analyses (Table 3). Furthermore, calibration curves for the predictive model in both cohorts indicated that the predicted 3-month survival values were similar to the actual 3-month survival rates (Figure 4C and 4D). We then assessed the predictive accuracy of the prognostic model; the Harrell’s c-index values for the nomogram in the training and test cohorts were 0.684 and 0.670, respectively (*P*<0.01).

### Discussion

Using the training and test cohort patients in Nanjing City with AIS, we confirmed a correlation between low TG/HDL-C and increased case-fatality at 3 months, and this correlation was further supported by multivariate analyses. The discriminatory ability of TG/HDL-C for 3-month survival was superior to that of the other lipid measures. The established nomogram also explained the potential significance of TG/HDL-C in the prediction of AIS prognosis.

Atherothrombosis is a major subtype of TOAST classification system. A previous study reported that the percentages of the TOAST subtypes were as follows: atherothrombosis, 12% to 54%; cardioembolism, 10% to 26%; small-vessel, 20% to 42%; and undetermined/unclassified, 4% to 34% [16]. Therefore, there are different distributions of ischemic stroke subtypes among the Chinese population. The probable reason for this finding is the different study populations. In the present study, the most common stroke etiology was still atherothrombosis, which was similar to previous studies [17, 18]; however, many studies have shown that AIS in the Chinese population includes a large proportion of small-vessel occlusion [19]. This difference in subtypes occurred during a period of rapid economic development and changing lifestyles in China including changes in dietary fat and cholesterol intake.

The identification of a simple and inexpensive indicator for predicting disease prognosis is a global focus. The TG/HDL-C value can be reproduced and inexpensively determined during routine clinical management, and this study further confirms previous evidence of the clinical significance of its ability to predict prognosis. Emerging evidence has revealed that TG/HDL-C is correlated with insulin resistance [20] and predicts the incidence and mortality of cardiovascular disease [21]. Additionally, TG/HDL-C has a similar predictive ability for prognoses in hypertension [22] and diabetes mellitus [23]. Our prior study showed that TG/HDL-C could predict mortality and worse outcomes after AIS [13]. However, the cutoff of TG/HDL-C was determined with an receiver operating characteristic curve in our previous study [13]. We used X-tile software, a robust tool for outcome-based cutoff optimization, to determine the optimal cutoff value for TG/HDL-C (0.90) in the training cohort (Figure 1). However, compared to the previous cutoff value (0.87), which was determined by ROC analysis, the cutoff value of TG/HDL-C using X-tile software was a slightly higher in the same cohort of patients. Its discriminatory ability (sensitivity 82.5% and specificity 73.4%) was significantly greater than that in our prior study (sensitivity 67.8% and specificity 60.6%). Additionally, the discriminatory ability of TG/HDL-C was significantly greater than those of TG, TC, and their combination in both cohorts (Figure 3). Although smoking was clearly associated with TG/HDL-C, as shown in Tables S2 and S3, TG/HDL-C was an indicator that was independent of smoking in the training and test cohorts. Additionally, subgroup analyses by smoking status showed that in the univariate analysis, the TG/HDL-C ratio was significantly associated with 3-month mortality in the training cohort patients who smoked (*P*=0.014) and who did not smoke (*P*<0.001). Similar results were obtained for the test cohort patients (*P*=0.012 for smokers; *P*=0.010 for non-smokers). Collectively, we identified a favorable cutoff to predict 3-month case-fatality after AIS.

The nomogram is a visual and widely accepted approach to predict disease prognosis using several clinical characteristics [24]. Furthermore, emerging evidence has revealed the prognostic predictive ability of nomograms to be more precise than that of a traditional tumor staging system in malignancies [25, 26]. However, nomograms are rarely used for in stroke outcome. The current study attempted to develop a prognostic nomogram to predict 3-month mortality in the training and test cohort patients. Based on our nomogram, TG/HDL-C was included in the final model through a stepwise algorithm. The predictive effect of the nomogram was well explained by the calibration curve in both cohorts (Figures 4C and 4D). The results from nomogram showed that old age and a history of hypertension were also predictors of a poor prognosis in patients with AIS. Therefore, TG/HDL-C should be considered when predicting the prognosis of AIS.

A major strength of the present study is that it included a relatively large number of AIS patients from a single city; additionally, it involved both retrospective and prospective cohorts. However, additional studies should be conducted to confirm whether these results are applicable to other cohorts. Another strength of our study is the use of X-tile software, which is a robust graphics tool [27], to determine the optimal cutoffs for serum lipids. The imitations of the present study should be acknowledged when interpreting these findings. For example, the findings presented here are from individuals with AIS, limiting the extrapolation of the results to patients with hemorrhagic stroke. Validation for hemorrhagic stroke is required. Additionally, TG/HDL-C was included in the final model after Cox regression analysis, and we cannot confirm the dynamic changes in TG/HDL-C during treatment, and whether these dynamic changes influence the prognosis of AIS.

TG/HDL-C may be an objective parameter that can be easily obtained from routine laboratory estimates. In this study of 1459 AIS patients, TG/HDL-C was validated as a prognostic tool for 3-month survival after AIS. A cutoff value of TG/HDL-C of 0.9 in 1006 individuals, generated the most prognostic dichotomization of the retrospective and prospective cohorts regarding 3-month case fatality. These results suggest that TG/HDL-C, in combination with age and hypertension history, might improve the prediction of outcomes among AIS patients.

### Supplemetary Material

The Supplemenatry material for this article can be found online at:
www.aginganddisease.org/EN/10.14336/AD.2017.0629
**Supplemental table 1**. Other baseline bivariate comparison of training and test cohort.
**Supplemental table 2**. The association of clinical characteristics with lipid measures in the training cohort.
**Supplemental table 3**. The association of clinical characteristics with lipid measures in the test cohort.
