# Supplementary material for: The Short-term Prognostic Value of the Triglyceride-to-high-density Lipoprotein Cholesterol Ratio in Acute Ischemic Stroke
Source: Aging Dis. 2018 Jun 1;9(3):498–506. doi: 10.14336/AD.2017.0629 (PMC5988604; doi:10.14336/AD.2017.0629)
Supplement: Supplementary file 1 — The Supplemenatry material for this article can be found online at: www.aginganddisease.org/EN/10.14336/AD.2017.0629 Supplemental table 1. Other baseline bivariate comparison of training and test cohort. Supplemental table 2. The association of clinical characteristics with lipid measures in the training cohort. Supplemental table 3. The association of clinical characteristics with lipid measures in the test cohort. [file ad-9-3-498-s001.zip › ad-9-3-498-s001/ad-9-3-498-s001.pdf]

SUPPLEMENTARY DATA

# The Short-term Prognostic Value of the Triglyceride-to-high-density Lipoprotein Cholesterol Ratio in Acute Ischemic Stroke

**Qi-Wen Deng[#], Shuo Li[#], Huan Wang, Lei Zuo, Han-Qing Zhang, Zheng-Tian Gu, Fang-Lan Xing, Fu-Ling Yan***

Department of Neurology, Affiliated ZhongDa Hospital, School of Medicine, Southeast University, Nanjing, China.

© 2017. Deng Q et al. Published online at http://www.aginganddisease.org/EN/10.14336/AD.2017.0629

# SUPPLEMENTARY DATA

**Supplemental table 1.** Other baseline bivariate comparison of training and test cohort

| Demographic characteristics | Training cohort | | | | Test cohort | | | |
|---|---|---|---|---|---|---|---|---|
| | **Total** | **Incident alive** | **Incident death** | ***P* value [a]** | **Total** | **Incident death** | | ***P* value [a]** |
| Clinical characteristics | | | | | | | | |
| SBP (mm Hg) | 156 (140-171) | 155 (140-171) | 160 (150-173) | 0.058 | 155 (140-170) | 155 (140-169) | 162 (151-179) | 0.080 |
| DBP (mm Hg) | 86 (78-95) | 86 (78-95) | 86 (78-94) | 0.510 | 88 (80-96) | 88 (80-96) | 89 (81.5-94) | 0.982 |
| Medical history | | | | | | | | |
| Hypertension | 588 (58.4) | 519 (57.9) | 69 (63.3) | 0.276 | 277 (61.1) | 249 (60.4) | 28 (68.3) | 0.325 |
| Diabetes mellitus | 269 (26.7) | 235 (26.2) | 34 (31.2) | 0.266 | 118 (26.0) | 106 (25.7) | 12 (29.3) | 0.622 |
| History of atrial fibrillation | 223 (22.2) | 206 (23.0) | 17 (15.6) | 0.080 | 104 (23.0) | 96 (23.3) | 8 (19.5) | 0.582 |
| History of TIA | 26 (2.6) | 24 (2.7) | 2 (1.8) | 0.601 | 12 (2.6) | 10 (2.4) | 2 (4.7) | 0.390 |
| Stroke etiology [b] | | | | | | | | |
| Atherothrombosis | 753 (74.9) | 665 (74.2) | 88 (84.7) | 0.487 | 276 (60.9) | 252 (61.2) | 24 (58.5) | 0.434 |
| Cardioembolic | 153 (15.2) | 140 (15.6) | 13 (11.9) | | 83 (18.3) | 78 (18.9) | 5 (12.2) | |
| Small-vessel | 58 (5.8) | 54 (6.0) | 4 (3.7) | | 44 (9.7) | 39 (9.5) | 5 (12.2) | |
| Undetermined/unclassified | 42 (4.2) | 38 (4.2) | 4 (3.7) | | 50 (11.0) | 43 (10.4) | 7 (17.1) | |
| Laboratory characteristics | | | | | | | | |
| WBC ($10^9$/L) | 7.62 (6.02-9.53) | 7.60 (6.01-9.53) | 7.79 (6.10-9.93) | 0.630 | 7.60 (5.90-9.41) | 7.59 (5.83-9.41) | 7.62 (5.99-9.38) | 0.903 |
| Platelet ($10^9$/L) | 180 (147-215) | 179 (146-215) | 189 (149-209) | 0.709 | 182 (145-212) | 181 (142-211) | 184 (150-230) | 0.507 |
| Glucose (mmol/L) | 6.39 (5.28-8.07) | 6.36 (5.28-8.05) | 6.85 (5.32-8.19) | 0.095 | 6.37 (5.21-8.02) | 6.36 (5.20-8.08) | 6.63 (5.25-7.58) | 0.934 |
| Creatinine (mmol/L) | 75 (63-88) | 75 (63-89) | 72 (62-81) | 0.728 | 75 (64-91) | 74.5 (64-91) | 76 (64-84) | 0.630 |

SBP, systolic blood pressure; DBP, diastolic blood pressure; TIA, transient ischemic attack; WBC, white blood cell;
Values are medians (interquartile range) or frequencies and percentages.
[a] $\chi^2$ test or Mann-Whitney *U* test.
[b] According to the modified TOAST classification.

© 2017. Deng Q et al. Published online at http://www.aginganddisease.org/EN/10.14336/AD.2017.0629

# SUPPLEMENTARY DATA

**Supplemental table 2.** The association of clinical characteristics with lipid measures in the training cohort.

| **Demographic characteristics** | **Lipid measures** | | | | | | | | |
|---|---|---|---|---|---|---|---|---|---|
| | **TG** | | | **TC** | | | **TG/HDL-C** | | |
| | **Low** | **High** | ***P*** [a] | **Low** | **High** | ***P*** [a] | **Low** | **High** | ***P*** [a] |
| Age | 70 (58-79) | 68 (57-76) | 0.223 | 70 (59-77) | 68 (57-76) | 0.128 | 70 (58-78) | 67 (57-75) | 0.562 |
| Gender (male) | 90 (76.9) | 495 (55.7) | **<0.001** | 186 (34.9) | 399 (57.3) | 0.428 | 268 (68.9) | 317 (51.4) | **<0.001** |
| BMI (kg/m$^2$) | 23.8 (21.0-24.4) | 24.0 (22.1-24.8) | 0.152 | 23.2 (22.1-24.6) | 24.1 (21.9-25.2) | 0.128 | 23.6 (21.4-25.0) | 24.3 (22.0-25.2) | 0.422 |
| Clinical characteristics | | | | | | | | | |
| Smoking | 46 (39.3) | 199 (22.4) | **<0.001** | 81 (26.1) | 164 (23.6) | 0.381 | 135 (34.7) | 110 (17.8) | **<0.001** |
| Baseline NIHSS | 7 (3-17) | 8 (3-16) | 0.643 | 8 (3-17) | 8 (3-16) | 0.789 | 8 (3-17) | 8 (3-16) | 0.100 |
| Systolic blood pressure (mm Hg) | 155 (143-168) | 156 (140-172) | 0.524 | 155 (144-168) | 156 (140-173) | 0.700 | 155 (141-167) | 157 (140-176) | 0.147 |
| Diastolic blood pressure (mm Hg) | 88 (79-98) | 86 (78-95) | 0.332 | 87 (78-95) | 86 (78-95) | 0.695 | 87 (79-94) | 85 (78-96) | 0.562 |
| Medical history | | | | | | | | | |
| Hypertension | 98 (83.8) | 668 (75.1) | **0.040** | 243 (78.4) | 523 (75.1) | 0.265 | 320 (82.3) | 446 (72.3) | **<0.001** |
| Diabetes mellitus | 42 (35.9) | 227 (25.5) | **0.017** | 94 (30.3) | 175 (25.1) | 0.087 | 126 (32.4) | 143 (23.2) | **0.001** |
| History of atrial fibrillation | 7 (6.0) | 216 (24.3) | **<0.001** | 59 (19.0) | 164 (23.6) | 0.110 | 64 (16.5) | 159 (25.8) | **<0.001** |
| History of TIA | 3 (2.6) | 23 (2.6) | **<0.001** | 7 (2.3) | 19 (2.7) | 0.663 | 12 (3.1) | 14 (2.3) | 0.427 |
| Stroke etiology [b] | | | | | | | | | |
| Atherothrombosis | 73 (62.4) | 680 (76.5) | **<0.001** | 221 (71.3) | 532 (76.4) | 0.359 | 287 (73.8) | 466 (75.6) | 0.824 |
| Cardioembolic | 16 (13.7) | 137 (15.4) | | 53 (17.1) | 100 (14.4) | | 60 (15.4) | 93 (15.1) | |
| Small-vessel | 17 (14.5) | 41 (4.6) | | 20 (6.5) | 38 (5.5) | | 23 (5.9) | 35 (5.7) | |
| Undetermined/unclassified | 11 (9.4) | 31 (3.5) | | 16 (5.2) | 26 (3.7) | | 19 (4.9) | 23 (3.7) | |
| Therapy of thrombolysis | 12 (10.3) | 89 (10.0) | 0.934 | 35 (11.3) | 66 (9.5) | 0.378 | 41 (10.5) | 60 (9.7) | 0.675 |
| Endovascular intervention | 8 (6.8) | 34 (3.8) | 0.126 | 20 (5.8) | 22 (3.4) | 0.084 | 17 (4.4) | 25 (4.1) | 0.806 |
| Laboratory characteristics | | | | | | | | | |
| WBC (10$^9$/L) | 6.85 (5.43-8.71) | 7.72 (6.10-9.61) | **0.001** | 7.69 (6.19-9.69) | 7.61 (5.97-9.41) | 0.378 | 7.74 (6.00-9.73) | 7.58 (6.06-9.41) | 0.922 |
| Platelet (10$^9$/L) | 191 (155-209) | 178 (146-217) | 0.883 | 186 (149-222) | 177 (146-213) | 0.154 | 190 (149-224) | 176 (146-211) | 0.096 |
| Glucose (mmol/L) | 7.46 (6.58-8.48) | 6.18 (5.18-7.97) | **<0.001** | 6.55 (5.36-8.27) | 6.22 (5.25-7.91) | 0.058 | 6.58 (5.32-8.23) | 6.11 (5.21-7.69) | 0.378 |
| Creatinine (mmol/L) | 72 (63-89) | 75 (63-88) | 0.446 | 74 (62-89) | 75 (63-87) | 0.977 | 74 (64-87) | 75 (62-91) | 0.096 |

BMI, body mass index; NIHSS, NIH Stroke Scale; TIA, transient ischemic attack; WBC, white blood cell; TG, Triglycerides; TC, total cholesterol; HDL-C, high-density lipoprotein cholesterol; TG/HDL-C, TG to HDL-C ratio.
Values are medians (interquartile range) or frequencies and percentages.
Individual patients were divided into low (<cutoff value) and high (≥cutoff value) level groups according to corresponding cutoffs of TG, TC, and TG/HDL-C.
Statistically significant results were in bold.
[a] $\chi^2$ test or Mann-Whitney *U* test.
[b] According to the modified TOAST classification.

© 2017. Deng Q et al. Published online at http://www.aginganddisease.org/EN/10.14336/AD.2017.0629

# SUPPLEMENTARY DATA

**Supplemental table 3.** The association of clinical characteristics with lipid measures in the test cohort.

| **Demographic characteristics** | **Lipid measures** | | | | | | | | |
|---|---|---|---|---|---|---|---|---|---|
| | **TG** | | | **TC** | | | **TG/HDL-C** | | |
| | **Low** | **High** | ***P*** [a] | **Low** | **High** | ***P*** [a] | **Low** | **High** | ***P*** [a] |
| Age | 71.5 (60-79) | 68 (56-76) | 0.064 | 71 (59-77) | 67 (56-76) | 0.090 | 70 (59-78) | 67 (55-75) | 0.075 |
| Gender (male) | 46 (76.7) | 222 (76.7) | **0.003** | 87 (64.4) | 181 (56.9) | 0.136 | 127 (70.9) | 141 (51.4) | **<0.001** |
| BMI (kg/$m^2$) | 23.1 (20.2-25.1) | 23.8 (21.4-25.4) | 0.149 | 23.0 (21.1-24.8) | 24.0 (21.2-25.6) | 0.282 | 23.3 (21.2-24.9) | 24.4 (20.8-25.6) | 0.229 |
| Clinical charactersitics | | | | | | | | | |
| Smoking | 23 (38.3) | 95 (23.4) | 0.020 | 42 (31.1) | 76 (23.9) | 0.110 | 65 (36.3) | 53 (19.3) | **<0.001** |
| Baseline NIHSS | 7 (3-17) | 9 (3-16) | 0.719 | 9 (3-18) | 8.5 (3-16) | 0.719 | 8 (3-17) | 9 (3-16) | 0.719 |
| SBP (mm Hg) | 155 (145-168) | 155 (140-171) | 0.521 | 155 (140-165) | 156 (140-174) | 0.521 | 155 (142-165) | 156 (140-176) | 0.521 |
| DBP (mm Hg) | 88.5 (79-98) | 88 (80-95.5) | 0.786 | 89 (80-98) | 88 (80-95) | 0.786 | 89 (79-94) | 87.5 (80-98) | 0.786 |
| Medical history | | | | | | | | | |
| Hypertension | 41 (68.3) | 236 (60.1) | 0.220 | 85 (63.0) | 192 (60.4) | 0.606 | 119 (66.5) | 158 (57.7) | 0.059 |
| Diabetes mellitus | 19 (31.7) | 99 (25.2) | 0.287 | 40 (29.7) | 78 (24.5) | 0.258 | 55 (30.7) | 63 (23.0) | 0.067 |
| History of AT | 3 (5.0) | 101 (25.7) | **<0.001** | 23 (17.0) | 81 (25.5) | 0.051 | 27 (15.1) | 77 (28.1) | **0.001** |
| History of TIA | 2 (3.3) | 10 (2.5) | 0.723 | 3 (2.3) | 9 (2.8) | 0.712 | 6 (3.14) | 6 (2.2) | 0.451 |
| Stroke etiology [b] | | | | | | | | | |
| Atherothrombosis | 37 (61.7) | 239 (60.8) | 0.652 | 88 (65.2) | 188 (59.1) | 0.372 | 107 (59.8) | 169 (61.7) | 0.080 |
| Cardioembolic | 13 (21.7) | 70 (17.8) | | 23 (17.0) | 60 (18.9) | | 42 (23.5) | 41 (15.0) | |
| Small-vessel | 6 (10.0) | 38 (9.7) | | 14 (10.4) | 30 (9.4) | | 15 (8.4) | 29 (10.6) | |
| Undetermined/unclassified | 4 (6.7) | 46 (11.7) | | 10 (7.4) | 40 (12.6) | | 15 (8.4) | 35 (12.8) | |
| Therapy of thrombolysis | 2 (3.3) | 33 (8.4) | 0.171 | 13 (9.6) | 22 (6.9) | 0.323 | 15 (8.4) | 20 (7.3) | 0.674 |
| Endovascular intervention | 2 (3.3) | 11 (2.8) | 0.817 | 5 (3.7) | 8 (2.5) | 0.489 | 7 (3.9) | 6 (2.2) | 0.284 |
| Laboratory characteristics | | | | | | | | | |
| WBC ($10^9$/L) | 7.04 (5.49-8.71) | 7.60 (6.00-9.52) | **0.035** | 7.72 (6.21-9.69) | 7.55 (5.75-9.04) | 0.079 | 7.78 (5.70-9.73) | 7.42 (5.96-9.04) | 0.079 |
| Platelet ($10^9$/L) | 191 (155-213) | 178 (141-212) | 0.119 | 186 (147-222) | 177 (145-210) | 0.119 | 190 (147-223) | 175 (144-209) | 0.119 |
| Glucose (mmol/L) | 7.46 (6.63-8.48) | 6.06 (5.11-7.82) | **<0.001** | 6.58 (5.31-8.27) | 6.20 (5.14-7.69) | **0.008** | 6.92 (5.35-8.19) | 6.07 (5.12-7.58) | **<0.001** |
| Creatinine (mmol/L) | 73 (64-89) | 75 (64-91) | 0.935 | 76 (64-91) | 74 (64-87.5) | 0.916 | 76 (65-89) | 73 (63-91) | 0.935 |

BMI, body mass index; NIHSS, NIH Stroke Scale; TIA, transient ischemic attack; WBC, white blood cell; TG, Triglycerides; TC, total cholesterol; HDL-C, high-density lipoprotein cholesterol; TG/HDL-C, TG to HDL-C ratio.

Values are medians (interquartile range) or frequencies and percentages.

Individual patients were divided into low (<cutoff value) and high (≥cutoff value) level groups according to corresponding cutoffs of TG, TC, and TG/HDL-C.

Statistically significant results were in bold.

[a] $\chi^2$ test or Mann-Whitney *U* test.

[b] According to the modified TOAST classification.

© 2017. Deng Q et al. Published online at http://www.aginganddisease.org/EN/10.14336/AD.2017.0629
